# Serum fibrinogen-like protein 2 is associated with diabetic nephropathy severity and modulates high glucose-induced tubular dysfunction via Akt-FoxO1 signaling

**DOI:** 10.1080/0886022X.2026.2672189

**Published:** 2026-05-20

**Authors:** Xiao Gang Lu, Hui Jin, Ya Pei Wang, Hong Bin Li, Yin Qin Cheng

**Affiliations:** a Department of Clinical Nutrition, Nantong Second People’s Hospital, Nantong City, Jiangsu, China; b Department of Endocrinology and Metabolism, Nantong Second People’s Hospital, Nantong City, Jiangsu, China

**Keywords:** Fibrinogen-like 2 (FGL2), diabetic nephropathy, renal tubular cells, PI3K/Akt-FoxO1 signaling, high-glucose-induced injury

## Abstract

Diabetic nephropathy (DN) is a major cause of chronic kidney disease, and sensitive biomarkers of early tubular injury remain limited. Fibrinogen-like protein 2 (FGL2) has been implicated in inflammation and fibrosis, but its clinical significance and mechanistic role in DN are unclear. Serum FGL2 levels were measured in 105 patients with T2DM stratified by albuminuria and in 110 healthy controls, and its associations with metabolic parameters, renal injury, inflammation, and fibrosis were analyzed. *In vitro*, HK-2 cells were exposed to high glucose, and FGL2 was silenced using siRNA to assess effects on PI3K/Akt-FoxO1 signaling, cell viability, apoptosis, oxidative stress, and ECM remodeling. Pathway specificity was confirmed using a PI3K/Akt inhibitor. Serum FGL2 levels were higher in patients with T2DM than in controls and increased progressively with the severity of albuminuria. Circulating FGL2 positively correlated with glycemic indices, insulin resistance, lipid parameters, renal tubular injury markers (NGAL, KIM-1), inflammatory cytokines (TNF-α, IL-6), and fibrotic mediators (TGF-β1, CTGF). High glucose induced FGL2 expression in HK-2 cells in a dose-dependent manner. Silencing FGL2 enhanced Akt and FoxO1 phosphorylation, improved cell viability, reduced apoptosis, attenuated oxidative stress, restored antioxidant enzyme activity, and suppressed ECM-related gene and protein expression. These protective effects were reversed by PI3K/Akt inhibition. Serum FGL2 is elevated in patients with T2DM and correlates with the severity of renal injury, while mechanistically contributing to high glucose-induced tubular dysfunction *via* the PI3K/Akt-FoxO1 signaling pathway.

## Key Messages

**What is known?**

- Current clinical markers mainly reflect glomerular damage, whereas sensitive and reliable biomarkers for early tubular dysfunction are limited.
- FGL2 is involved in inflammatory and fibrotic processes in several diseases; however, its relevance to DN and renal tubular injury remains incompletely elucidated.

**What does this study add?**

- This study demonstrated that serum FGL2 levels were significantly elevated in patients with T2DM and progressively increased with worsening albuminuria.
- Circulating FGL2 levels are closely associated with metabolic dysregulation, renal tubular injury markers (NGAL and KIM-1), inflammation, and renal fibrosis.
- Mechanistically, FGL2 directly contributes to high-glucose-induced tubular epithelial injury by modulating the PI3K/Akt-FoxO1 signaling pathway, thereby promoting oxidative stress, apoptosis, and extracellular matrix remodeling.

**What is the potential impact?**

- FGL2 may serve as a novel biomarker for the early detection and assessment of the severity of tubular injury in DN.
- Targeting FGL2 or its downstream PI3K/Akt-FoxO1 signaling axis could represent a promising therapeutic strategy to prevent or slow the progression of DN.
- These findings provide new insights into the molecular mechanisms linking metabolic stress to tubular dysfunction, with potential implications for risk stratification and personalized interventions in DKD.

## Graphical abstract

**Figure:**
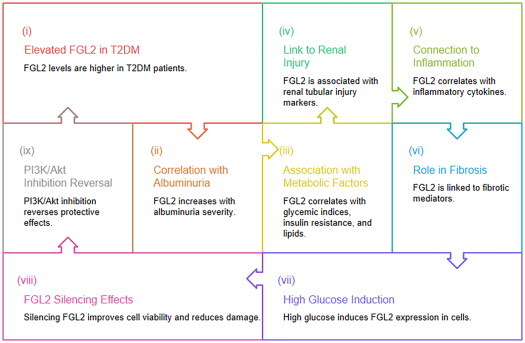

Schematic illustration of the multifaceted involvement of fibrinogen-like protein 2 (FGL2) in type 2 diabetes mellitus (T2DM) and diabetic kidney disease (DKD). Circulating FGL2 levels are elevated in patients with T2DM and progressively increase with worsening albuminuria. Increased FGL2 is closely associated with metabolic dysregulation, including impaired glycemic control, insulin resistance, and dyslipidemia. Within the kidney, FGL2 expression correlates with markers of tubular injury, pro-inflammatory cytokine production, and activation of profibrotic mediators. Mechanistically, high-glucose conditions induce FGL2 expression in renal cells, whereas FGL2 silencing enhances cell viability and mitigates cellular injury. These protective effects are abolished by inhibition of the PI3K/Akt signaling pathway. Collectively, the schematic highlights FGL2 as a key mediator linking metabolic dysfunction to inflammatory and fibrotic pathways that drive diabetic renal injury.

## Introduction

Diabetic kidney disease (DKD) is the predominant cause of end-stage kidney disease worldwide and severely affects the morbidity and mortality of individuals with diabetes. Emerging epidemiological data suggest that approximately 30–40% of patients with type 1 or type 2 diabetes will develop DKD, and the condition accounts for a large fraction of the global chronic kidney disease (CKD) burden [1,2]. Historically, research and clinical attention have focused on glomerular lesions (including glomerular hyperfiltration, basement membrane thickening, mesangial expansion, podocyte loss, and glomerulosclerosis) as drivers of DKD progression. However, in recent years, it has become increasingly clear that renal tubular epithelial injury and tubulointerstitial disease are critical, early, and independent contributors to the progression of DKD. Tubular cell dysfunction, manifesting as increased oxidative stress, mitochondrial injury, endoplasmic reticulum stress, inflammation, epithelial-to-mesenchymal transition (EMT), and cellular senescence, plays a central role in driving tubulointerstitial fibrosis, interstitial inflammation, capillary rarefaction, and declining renal function [3,4].

Given the complex and multifactorial pathogenesis of DKD, there is an urgent need for robust circulating biomarkers that reflect early tubular injury, provide mechanistic insights, and may serve as therapeutic targets. Although albuminuria and estimated glomerular filtration rate (eGFR) are long-standing clinical indicators of renal damage, they are imperfect indicators. Microalbuminuria may result in the failure to detect early tubular injury, and by the time albuminuria appears, substantial injury may have already occurred. Thus, the search for novel biomarkers, especially those that signal tubular pathology and are directly linked to mechanistic pathways, is an active area of research [5,6].

Fibrinogen-like protein 2 (FGL2) is a multifunctional protein expressed in immune cells, endothelial cells, and certain parenchymal tissues. It exists in both a membrane-bound form (mFGL2) with prothrombinase/coagulation activity and a soluble form (sFGL2) with immunomodulatory functions [7]. FGL2 has been implicated in inflammatory, procoagulant, and fibrotic disorders in multiple organs. Transcriptomic analyses of the kidney have revealed that FGL2 mRNA is upregulated in human CKD and is associated with worse fibrotic outcomes [8]. Moreover, earlier experimental work in diabetic nephropathy models demonstrated that FGL2 expression correlates with renal microthrombosis and ischemia [8]. A recent review highlighted FGL2 as a key mediator of inflammation and coagulation in renal disease and suggested that it may serve as both a biomarker and a mechanistic driver of renal injury [7].

Despite these advances, important gaps remain. First, human studies have not directly established whether circulating FGL2 reflects tubular injury versus glomerular damage. Serum FGL2 may be derived from multiple cell types, including immune cells, endothelial cells, and tubular epithelial cells [9,10]. To address this, mechanistic studies in renal tubular epithelial cells are critical to determine whether FGL2 is upregulated in response to high glucose–induced tubular stress and contributes to tubular dysfunction. Our study combines clinical measurements of circulating FGL2 with functional experiments in human tubular cells to explore this link, providing insight into its potential role as a marker of tubular stress and profibrotic signaling in DKD.

Hyperglycemia-induced maladaptive signaling in tubular epithelial cells is a hallmark of DKD, and the phosphatidylinositol-3-kinase (PI3K)/protein kinase B (Akt) pathway and its downstream target, forkhead box O1 (FoxO1), are key mediators. FoxO1 regulates genes that control oxidative stress, autophagy, the cell cycle, apoptosis, and metabolic homeostasis. High-glucose conditions lead to Akt hyperactivation, resulting in FoxO1 phosphorylation and nuclear exclusion. This creates a positive feedback loop in which Akt activation suppresses FoxO1, further amplifying pro-mTORC1 signaling, oxidative stress, and cellular injury [11,12]. In tubular cells, FoxO1 overexpression mitigates mitochondrial dysfunction and epithelial injury in diabetic models [13].

Based on these clinical and mechanistic insights, we hypothesized that (i) serum FGL2 levels are elevated in patients with DKD and (ii) FGL2 directly modulates tubular epithelial cell responses to high glucose *via* the Akt-FoxO1 signaling pathway, contributing to tubular dysfunction and a profibrotic milieu in DKD. To test these hypotheses, we measured circulating FGL2 in diabetic cohorts stratified by nephropathy status and investigated the effects of FGL2 manipulation in human renal tubular epithelial cells under high-glucose stress, assessing Akt activation, FoxO1 localization and activity, oxidative stress, apoptosis, mitochondrial function, and fibrotic gene expression.

## Materials and methods

### Study population

**Figure 1.**
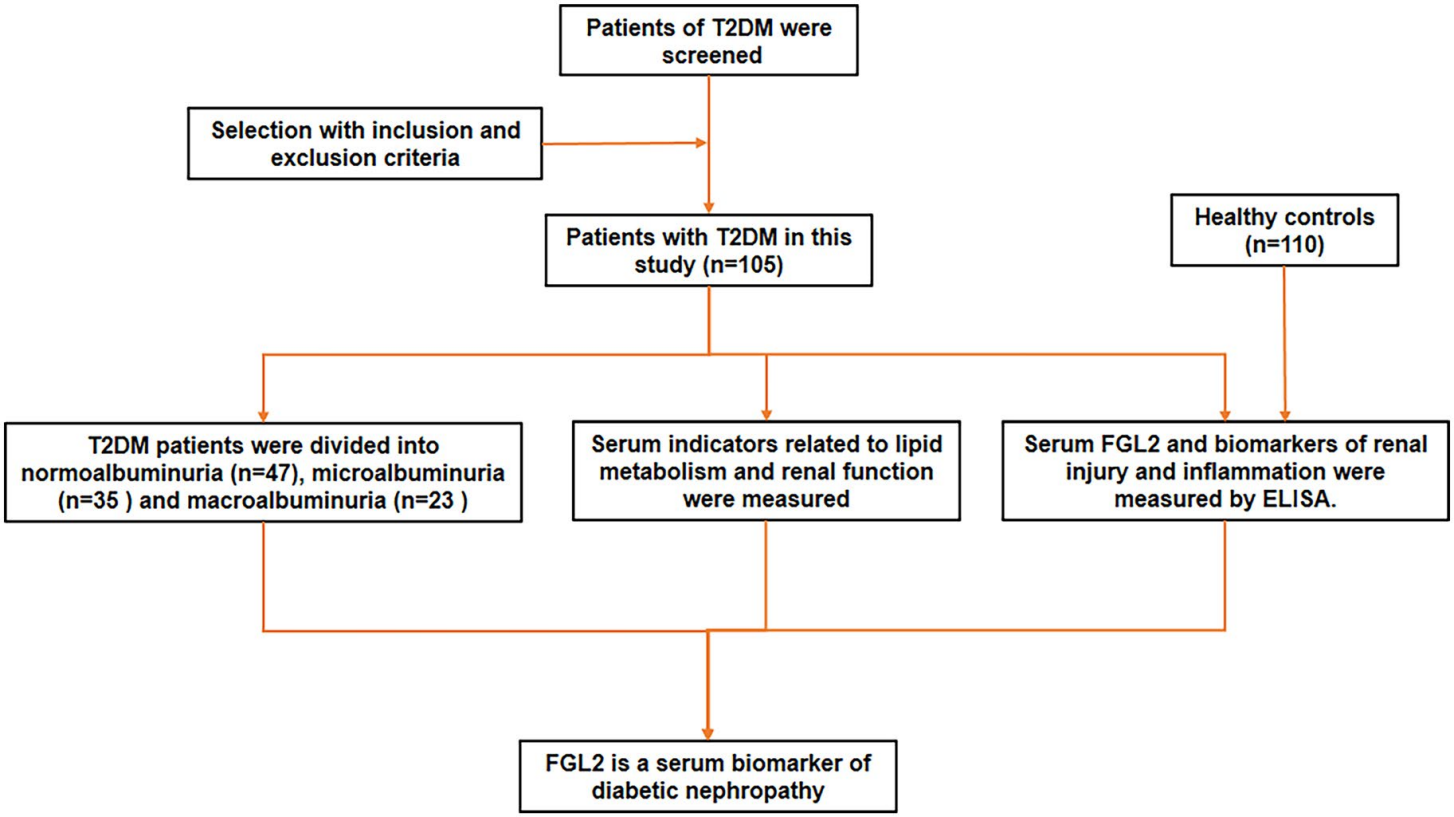
Flow chart of clinical study for patient screening, measurement and analysis.

This cross-sectional study included 105 patients with T2DM who were admitted to the Department of Endocrinology of our hospital between January 2023 and June 2025. T2DM was diagnosed according to the following criteria: a fasting glucose ≥ 7.0 mmol/L or a glycosylated hemoglobin (HbA1c) level of ≥ 6.5 mmol/L [14]. Based on the 24-h urinary albumin excretion rate (UAER), all 105 patients with T2DM were divided into three subgroups: normoalbuminuria (urine albumin-to-creatinine ratio (ACR) < 30 mg/g) (*n* = 47), microalbuminuria (30 ≤ ACR ≤ 300 mg/g) (*n* = 35), and macroalbuminuria (ACR > 300 mg/g) (*n* = 23) [15]. Urine was collected at 24 h and immediately examined to obtain the ACR. Diabetic nephropathy was defined as the presence of microalbuminuria or macroalbuminuria. We also selected 110 subjects who underwent health examinations as the control group, matched for age and sex. The exclusion criteria were as follows: (i) type 1 diabetes, (ii) infectious disease, (iii) history of malignancy, (iv) liver cirrhosis, (v) acute and chronic nephritis, and (vi) cardiovascular disorders. This study protocol was approved by the Research Ethics Committee of our hospital. Figure 1 summarizes the clinical research process.

### Data collection

Basic population information and experimental measurements of the enrolled patients were collected after admission, including age, sex, T2DM duration, and the presence of hypertension. BMI was expressed as weight per square kilometer (kg/m^2^). Laboratory data were collected, including blood albumin, lymphocyte, fasting blood glucose (FBG), HbA1c, fasting insulin, triglyceride (TG), total cholesterol (TC), blood urea nitrogen (BUN), and serum creatinine (Scr) levels. Prognostic nutritional index (PNI) = Blood albumin (g/L) + 5 × lymphocyte count (mmol/L). HOMA-IR = FBG (mmol/L) × fasting insulin (μU/mL) ÷ 22.5.

### Measurement of serum biomarkers

Serum samples were collected, centrifuged at 2,000 × g for 10 min, and stored at −80 °C until analysis. Serum FGL2 concentrations of neutrophil gelatinase-associated lipocalin (NGAL; QK1757, R&D Systems), kidney injury molecule-1 (KIM-1; DSKM100, R&D Systems), tumor necrosis factor-alpha (TNF-α; SEKH-0047, Solarbio), interleukin-6 (IL-6; D6050B, R&D Systems), transforming growth factor-β1 (TGF-β1; SEKH-0316, Solarbio), connective tissue growth factor (CTGF; DY9190-05, R&D Systems), and FGL2 (ml063599, Shanghai Enzyme-linked Biotechnology) were measured using ELISA kits. All assays were performed in triplicates, according to the manufacturer’s instructions. Absorbance was measured at 450 nm using a microplate reader, and the concentrations were calculated from standard curves using a four-parameter logistic regression.

### Cell culture

Human renal tubular cells (HK-2, Cat. No. CRL-2190, American Type Culture Collection, Manassas, VA, USA) were cultured in Dulbecco’s modified Eagle’s medium (DMEM, Gibco, Cat. No. 11965-092, Thermo Fisher Scientific, USA) supplemented with 10% fetal bovine serum (FBS, Cat. No. 10270-106, Gibco, Thermo Fisher Scientific, USA) in a 37 °C, 5% CO_2_ incubator. To inhibit PI3K/Akt signaling, HK-2 cells were pretreated with the PI3K/Akt inhibitor LY294002 (10 μM, HY-10108, MedChemExpress) for 1 h, followed by exposure to high glucose (30 mM) for an additional 24 h.

### Cell grouping

For *in vitro* experiments, HK-2 cells were subjected to two experimental protocols. In the first protocol, cells were treated with increasing concentrations of D-glucose (5.5, 10, 20, 30, and 50 mM) for 48 h to evaluate the dose-dependent effects of high glucose exposure. In the second protocol, HK-2 cells were divided into four groups: (i) control group, (ii) model group (HG+si-NC), in which cells were transfected with negative control siRNA (si-NC) for 24 h and subsequently exposed to high glucose (30 mM) for an additional 24 h; (iii) FGL2 silencing group (HG+si-FGL2), in which cells were transfected with si-FGL2 for 24 h followed by high glucose treatment (30 mM) for 24 h; and (iv) pathway inhibitor group (HG+si-FGL2 + LY294002), in which cells were transfected with si-FGL2 for 24 h, pretreated with the PI3K inhibitor LY294002 (10 μM) for 1 h, and then exposed to high glucose (30 mM) for 24 h. This experimental design ensured efficient FGL2 knockdown while reliably modeling acute high-glucose-induced tubular epithelial injury and allowing mechanistic assessment of PI3K/Akt pathway involvement. Although osmotic control (mannitol-treated) groups are commonly used in high-glucose experimental systems, such a control was not included in the present study owing to experimental design constraints.

### Cell transfection

HK-2 cells in the logarithmic growth phase were seeded into 6-well plates at a density of 1.0 × 10^5^ cells per well and cultured until they reached 70–80% confluence. Cells were transfected with FGL2 siRNA (si-FGL2) or a negative control siRNA (si-NC) using Lipofectamine 2000 (Invitrogen, Carlsbad, CA, USA). To evaluate transfection efficiency under basal glucose conditions, cells were maintained for 48 h after transfection, and FGL2 knockdown was confirmed by RT-qPCR and western blot analysis. In contrast, for all subsequent functional assays, cells were transfected for 24 h, then exposed to high-glucose conditions for an additional 24 h, as described in the Cell Grouping section.

### Cell viability assay

Cell viability was assessed using the MTT assay (M1020; Solarbio, Beijing, China) according to the manufacturer’s protocol. Briefly, cells were seeded in 96-well plates at a density of 5 × 10³-1 × 10^4^ cells/well and treated as indicated. After treatment, 10 µL of MTT solution (5 mg/mL) was added to each well and incubated at 37 °C for 4 h. The supernatant was carefully removed, and 100 µL of dimethyl sulfoxide (DMSO, Cat. No. D2650, Sigma-Aldrich, St. Louis, MO, USA) was added to dissolve the formazan crystals. Absorbance was measured at 570 nm using a microplate reader (Bio-Rad, USA). Cell viability was expressed as a percentage of that in the control group, representing metabolic activity and cell proliferation. All experiments were performed in triplicate.

### Apoptosis assay

Apoptosis was evaluated using an Annexin V-FITC Apoptosis Detection Kit (CA1020; Solarbio, Beijing, China), according to the manufacturer’s instructions. Briefly, HK-2 cells were seeded in 6-well plates and subjected to the indicated treatments. After incubation, both adherent and floating cells were collected, washed twice with cold phosphate-buffered saline (PBS, Cat. No. 10010-031, Gibco, Thermo Fisher Scientific, USA), and resuspended in 1× binding buffer at a concentration of 1 × 10^6^ cells/mL. Subsequently, 5 µL of Annexin V-FITC and 5 µL of propidium iodide (PI, Cat. No. P4170, Sigma-Aldrich, USA) were added to 100 µL of the cell suspension and incubated for 15 min at room temperature in the dark. After adding 400 µL of binding buffer, the samples were immediately analyzed using a flow cytometer (BD FACSCanto II; BD Biosciences, USA). All assays were performed in triplicate.

### Immunofluorescence staining

Intracellular reactive oxygen species (ROS) generation was detected in HK-2 cells using dihydroethidium (DHE; S0063, Beyotime, Shanghai, China) according to the manufacturer’s protocol. Briefly, HK-2 cells were seeded onto sterile glass coverslips in 24-well plates and subjected to the indicated treatments. After washing twice with PBS, the cells were incubated with 10 µM DHE solution at 37 °C for 30 min in the dark. Following incubation, the cells were gently rinsed with PBS to remove excess dye and immediately visualized under a fluorescence microscope (Leica DMi8, Germany) at 200× magnification. The red fluorescence intensity, indicative of intracellular ROS levels, was quantified using the ImageJ software in at least five randomly selected fields per sample. All experiments were performed in triplicate.

### Oxidation stress analysis

HK-2 cells were harvested and washed twice with ice-cold PBS, then homogenized on ice at 10% (w/v) in ice-cold PBS using a glass/Teflon homogenizer. Homogenates were centrifuged at 10,000 × g for 10 min at 4 °C and the resulting supernatants were collected for biochemical assays. Malondialdehyde (MDA), superoxide dismutase (SOD) and catalase (CAT) activities were determined using commercial kits following the manufacturers’ protocols: MDA (S0131S, Beyotime, Shanghai, China), SOD (S0109, Beyotime, China) and CAT (S0051, Beyotime, China). All samples and standards were assayed in duplicate in 96-well plates, and the absorbance was measured using a microplate reader in accordance with the kit instructions. All experiments were independently repeated at least three times.

### Quantitative real-time PCR (RT-qPCR)

Total RNA was extracted from HK-2 cells using TRIzol reagent (Invitrogen, Carlsbad, CA, USA) in accordance with the manufacturer’s instructions. RNA purity and concentration were assessed by measuring absorbance at 260/280 nm using a NanoDrop spectrophotometer (Thermo Fisher Scientific, USA), and RNA integrity was verified using agarose gel electrophoresis. Complementary DNA (cDNA) was synthesized from 1 µg of total RNA using a reverse transcription kit (Takara, Japan) in accordance with the manufacturer’s protocol. Quantitative PCR was performed using a SYBR Green Master Mix (Takara, Japan) on a QuantStudio 6 Flex Real-Time PCR System (Applied Biosystems, USA). The cycling conditions were as follows: initial denaturation at 95 °C for 30 s, followed by 40 cycles at 95 °C for 5 s and 60 °C for 30 s. Relative gene expression levels were calculated using the 2^-ΔΔCt^ method, with GAPDH as the internal control. The primer sequences for all target genes are listed in Supplementary Table 1. Primer sequences for all target genes are listed in supplementary Table 1. Each reaction was performed in triplicate, and the experiments were independently repeated three times.

### Western blot analysis

Protein expression in HK-2 cells was analyzed using western blotting. Briefly, the cells were lysed in ice-cold RIPA buffer (Beyotime, Shanghai, China) containing protease and phosphatase inhibitors (Roche, Basel, Switzerland). The lysates were incubated on ice for 30 min, centrifuged at 12,000 × g for 15 min at 4 °C, and the supernatants were collected. Protein concentrations were determined using a BCA protein assay kit (Thermo Fisher Scientific, USA). Equal amounts of protein (50 µg) were separated by 10% SDS-PAGE and transferred onto polyvinylidene difluoride (PVDF) membranes (Millipore, Billerica, MA, USA). Membranes were blocked with 5% nonfat milk in Tris-buffered saline with 0.1% Tween-20 (TBST) for 1 h at room temperature and incubated overnight at 4 °C with the following primary antibodies: anti-FGL2 (1:500, sc-100276, rabbit monoclonal, Santa Cruz), anti-p-Akt (Ser473) (1:400, ab81283, rabbit monoclonal, Abcam), anti-Akt (1:400, ab8805, rabbit polyclonal, Abcam), anti-p-FoxO1 (1:500, ab131339, rabbit polyclonal, Abcam), anti-FoxO1 (1:500, ab39670, rabbit polyclonal, Abcam), and anti-GAPDH (1:2000, ab8245, mouse monoclonal, Abcam). After washing with TBST, the membranes were incubated for 1 h at room temperature with horseradish peroxidase-conjugated goat anti-rabbit or anti-mouse secondary antibodies (1:5000; Abcam). Protein bands were visualized using an enhanced chemiluminescence (ECL) detection kit (Thermo Fisher Scientific) and imaged using a ChemiDoc XRS+system (Bio-Rad, Hercules, CA, USA). Densitometric analysis was performed using the ImageJ software, and target protein levels were normalized to GAPDH. All experiments were independently repeated at least three times.

### Statistical analysis

Statistical analyses were performed using IBM SPSS Statistics (version 30.0) and GraphPad Prism (version 9.0). Normality was assessed with the Shapiro-Wilk test. Normally distributed variables are presented as mean ± standard deviation and compared using Student’s t-test or one-way ANOVA, whereas non-normally distributed variables are reported as median (interquartile range) and analyzed using the Mann–Whitney U test or Kruskal-Wallis test. Pearson’s or Spearman’s correlation coefficients were used according to data distribution, and P values were adjusted for multiple comparisons using the Benjamini-Hochberg false discovery rate (FDR) method. Multivariate logistic regression was conducted to identify independent risk factors for DN. All variables listed in Table 1, including lipid parameters (TG, TC) and inflammatory markers (TNF-α), were included in the multivariate model, along with age, sex, body mass index, duration of diabetes, and hypertension. Results are reported as odds ratios (ORs) with 95% confidence intervals (CIs). A two-tailed P value < 0.05 was considered statistically significant.

**Table 1.** Baseline characteristics of the included subjects.

Characteristics	Control (*n* = 110)	T2DM (*n* = 105)	*p* value
Age (years)	59.49 ± 8.26	58.74 ± 9.21	0.531
Sex (male, %)	62 (56.4%)	57 (54.3%)	0.759
BMI (kg/m^2^)	23.86 ± 4.45	24.47 ± 4.00	0.290
Hypertension (%)	39 (35.5%)	55 (52.4%)	0.012
Blood albumin (g/L)	41.33 ± 3.37	38.98 ± 3.90	<0.001
Lymphocyte (×10^9^/L)	2.25 ± 0.26	1.99 ± 0.26	<0.001
PNI	52.56 ± 3.56	48.91 ± 4.38	<0.001
FBG (mmol/L)	5.49 ± 0.61	7.72 ± 1.21	<0.001
HbA1c (%)	4.85 ± 0.47	7.77 ± 0.80	<0.001
Insulin (μU/mL)	8.97 ± 2.36	14.78 ± 4.70	<0.001
HOMA-IR	2.20 ± 0.68	5.16 ± 2.12	<0.001
TG (mmol/L)	1.42 ± 0.18	1.71 ± 0.23	<0.001
TC (mmol/L)	4.63 ± 0.54	5.43 ± 0.65	<0.001
BUN (mmol/L)	5.40 ± 0.64	7.04 ± 1.42	<0.001
Scr (μmol/L)	74.79 ± 8.24	94.28 ± 19.47	<0.001
NGAL (μg/mL)	82.38 ± 15.64	115.04 ± 25.82	<0.001
KIM-1 (ng/mL)	12.90 ± 1.62	20.08 ± 5.21	<0.001
TNF-α (pg/mL)	26.85 ± 4.27	39.98 ± 7.38	<0.001
IL-6 (pg/mL)	6.27 ± 0.86	9.12 ± 2.06	<0.001
TGF-β1 (pg/mL)	8.33 ± 1.18	11.93 ± 2.77	<0.001
CTGF (ng/mL)	3.74 ± 0.44	6.14 ± 1.29	<0.001

*Note:* Serum FGL2 levels are presented graphically in Figure 2.

Continuous variables are expressed as mean ± SD, and analyzed using t-test or Wilcoxon-Mann-Whitney test. Categorical variables are expressed as frequency (percentage), and analyzed using the chi-square test.

BMI: body mass index; PNI: prognostic nutritional index; FBG: fasting blood glucose; HOMA-IR: Homeostasis Model Assessment of Insulin Resistance; TG: triglyceride; TC: total cholesterol; BUN: blood urea nitrogen; Scr: serum creatinine; NGAL: neutrophil gelatinase-associated lipocalin; KIM-1: kidney injury molecule-1; TNF-α: tumor necrosis factor-α; IL-6: interleukin-6; TGF-β1: transforming growth factor-β1; CTGF: connective tissue growth factor.

## Results

### Clinical characteristics, correlation analyses, and independent risk factors for diabetic nephropathy

Baseline characteristics of the study population are summarized in Table 1. No significant differences were observed between the control and T2DM groups in age, sex, or BMI (all *p* > 0.05), whereas hypertension was significantly more prevalent in the T2DM group (*p* = 0.012). Compared with controls, individuals with T2DM had significantly lower serum albumin, lymphocyte counts, and prognostic nutritional index (PNI) (all *p* < 0.001), alongside markedly elevated fasting blood glucose (FBG), HbA1c, insulin, and HOMA-IR (all *p* < 0.001), indicating pronounced metabolic dysregulation and insulin resistance. In addition, lipid parameters (TG and TC), renal function markers (BUN and serum creatinine), and biomarkers of renal tubular injury, inflammation, and fibrosis, including NGAL, KIM-1, TNF-α, IL-6, TGF-β1, CTGF, and FGL2 were significantly higher in the T2DM group (all *p* < 0.001), suggesting an enhanced inflammatory burden and renal injury.

Further stratification of T2DM patients by albuminuria severity (Table 2) showed no significant differences in sex or BMI across subgroups; however, patients with macroalbuminuria were older and had a longer duration of diabetes than those with normoalbuminuria (*p* < 0.01). Nutritional and immune indicators, including serum albumin, lymphocyte count, and PNI, declined progressively with worsening albuminuria (all *p* < 0.01). Conversely, glycemic indices (FBG and HbA1c), insulin levels, and HOMA-IR increased significantly across disease stages (all *p* < 0.001), reflecting deteriorating metabolic control. Lipid parameters and renal dysfunction markers also rose in parallel with disease severity. Inflammatory cytokines, renal tubular injury markers, and fibrotic mediators showed stepwise increases from normoalbuminuria to macroalbuminuria (all *p* < 0.001).

**Table 2.** Baseline characteristics between all subgroups of T2DM.

Characteristics	Normoalbuminuria (*n* = 47)	Microalbuminuria (*n* = 35)	Macroalbuminuria (*n* = 23)	*p* value
Age (years)	55.57 ± 8.08	60.40 ± 9.12	62.70 ± 9.68	0.003
Sex (male, %)	24 (51.1%)	19 (54.3%)	14 (60.9%)	0.741
BMI (kg/m^2^)	24.34 ± 4.18	24.03 ± 4.41	25.39 ± 2.80	0.435
Duration (years)	8.16 ± 1.13	8.80 ± 1.52	9.58 ± 1.87	<0.001
Hypertension (%)	21 (44.7%)	19 (54.3%)	15 (65.2%)	0.261
Blood albumin (g/L)	40.69 ± 3.18	38.56 ± 3.78	36.12 ± 3.67	<0.001
Lymphocyte (×10^9^/L)	2.07 ± 0.28	1.95 ± 0.18	1.86 ± 0.25	0.002
PNI	51.06 ± 3.44	48.33 ± 4.10	45.42 ± 4.11	<0.001
FPG (mmol/L)	7.27 ± 1.01	7.78 ± 1.19	8.57 ± 1.16	<0.001
HbA1c (%)	7.48 ± 0.68	7.87 ± 0.76	8.24 ± 0.86	<0.001
Insulin (μU/mL)	11.79 ± 2.55	14.54 ± 2.90	21.24 ± 3.87	<0.001
HOMA-IR	3.81 ± 1.00	5.01 ± 1.20	8.13 ± 1.96	<0.001
TG (mmol/L)	1.64 ± 0.21	1.73 ± 0.24	1.80 ± 0.24	0.014
TC (mmol/L)	5.22 ± 0.56	5.53 ± 0.66	5.71 ± 0.70	0.006
BUN (mmol/L)	5.97 ± 0.73	7.40 ± 0.89	8.69 ± 1.30	<0.001
Scr (μmol/L)	82.92 ± 12.15	96.34 ± 17.02	114.34 ± 18.22	<0.001
NGAL (μg/mL)	96.16 ± 16.63	125.74 ± 18.96	137.34 ± 23.53	<0.001
KIM-1 (ng/mL)	15.98 ± 2.26	21.15 ± 2.77	26.84 ± 4.48	<0.001
TNF-α (pg/mL)	34.75 ± 4.58	42.14 ± 5.64	47.40 ± 6.38	<0.001
IL-6 (pg/mL)	7.72 ± 1.12	9.34 ± 1.45	11.64 ± 1.78	<0.001
TGF-β1 (pg/mL)	9.60 ± 1.35	12.87 ± 1.68	15.25 ± 1.88	<0.001
CTGF (ng/mL)	5.15 ± 0.64	6.41 ± 0.83	7.74 ± 1.01	<0.001

*Note:* Serum FGL2 levels are presented graphically in Figure 2.

BMI: body mass index; PNI: prognostic nutritional index; FBG: fasting blood glucose; HOMA-IR: Homeostasis Model Assessment of Insulin Resistance; TG: triglyceride; TC: total cholesterol; BUN: blood urea nitrogen; Scr: serum creatinine; NGAL: neutrophil gelatinase-associated lipocalin; KIM-1: kidney injury molecule-1; TNF-α: tumor necrosis factor-α; IL-6: interleukin-6; TGF-β1: transforming growth factor-β1; CTGF: connective tissue growth factor.

Using multivariate logistic regression (Table 3), we identified independent risk factors for DN after comprehensive adjustment for demographic characteristics, metabolic parameters, renal function indices, lipid profiles (TG and TC), inflammatory markers (TNF-α), and age, sex, BMI, diabetes duration, and hypertension. In this fully adjusted model, higher PNI was independently associated with a lower risk of diabetic nephropathy (OR = 0.775, 95% CI: 0.609–0.987, *p* = 0.039), suggesting a protective role of better nutritional status. In contrast, higher insulin resistance (HOMA-IR) was significantly associated with an increased risk of diabetic nephropathy (OR = 2.615, 95% CI: 1.221–5.599, *p* = 0.013). Among the renal function markers, BUN emerged as a strong independent risk factor (OR = 32.077, 95% CI: 3.264–315.214, *p* = 0.003), while serum creatinine showed a borderline association (*p* = 0.053). Importantly, elevated FGL2 levels remained significantly associated with DN (OR = 1.211, 95% CI: 1.039–1.411, *p* = 0.014) even after comprehensive adjustment for all covariates, including lipid profiles and inflammatory markers. Similarly, in an expanded model with further adjustment for TG, TC, and TNF-α, serum FGL2 continued to show a significant independent association with DN, confirming its robustness as a risk factor. Collectively, these findings indicate that elevated serum FGL2 is closely linked to metabolic dysregulation, renal tubular injury, inflammation, and fibrosis, and serves as an independent predictor of the development and progression of DN.

**Table 3.** Logistic multivariate regression for developing DN.

Characteristics	Odds ratio	95% confidence interval	*p* value
PNI	0.775	0.609–0.987	0.039
HOMA-IR	2.615	1.221–5.599	0.013
BUN (mmol/L)	32.077	3.264–315.214	0.003
Scr (μmol/L)	1.079	0.999–1.165	0.053
FGL2 (ng/mL)	1.211	1.039–1.411	0.014

### Serum FGL2 levels increased in patients with T2DM

**Figure 2.**
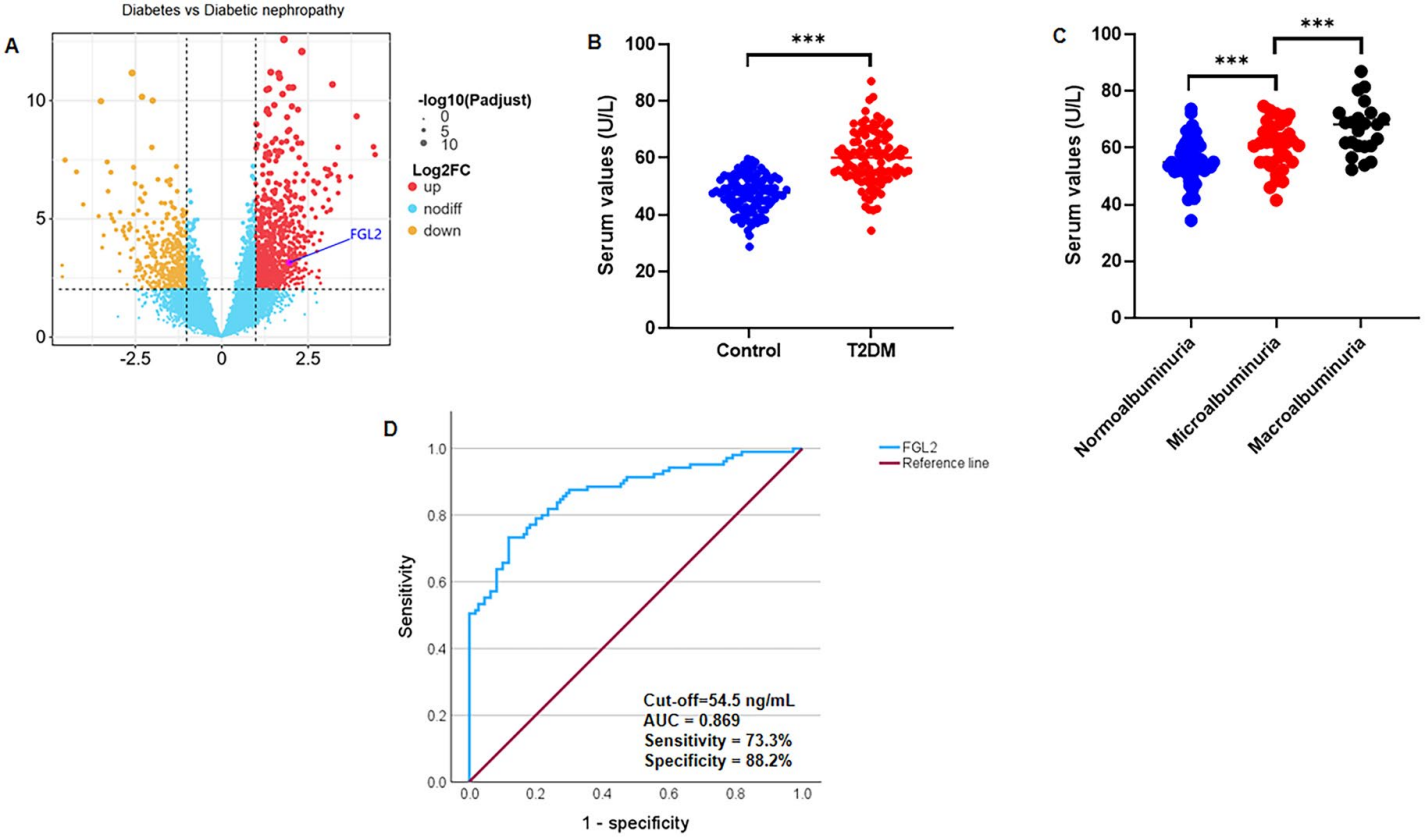
Circulating FGL2 is upregulated in T2DM and associated with the severity of diabetic nephropathy. (A) Volcano plot showing differentially expressed proteins between diabetes mellitus and diabetic nephropathy; FGL2 is highlighted as a significantly upregulated protein. The x-axis represents log2 fold change and the y-axis represents − log10 adjusted P value. (B) Comparison of serum FGL2 levels between healthy controls and T2DM patients. The serum FGL2 level in T2DM patients (*n* = 105) was significantly higher than that in healthy controls (*n* = 110). T test was applied. (C) Comparison of serum FGL2 level between three subgroups of T2DM patients. The serum FGL2 level in macroalbuminuria (*n* = 23) was significantly higher than that in normoalbuminuria (*n* = 47) and microalbuminuria (*n* = 35). ANOVA was applied. (D) The ROC curve was used to obtain the optimal cutoff value of serum FGL2 (54.5 ng/mL) that distinguishes the T2DM patients from healthy controls. ***p < 0.001.

To identify candidate biomarkers associated with diabetic nephropathy, differential expression analysis was first performed between diabetes mellitus and DN. As shown in Figure 2A, the volcano plot revealed a distinct expression profile, with FGL2 being significantly upregulated in DN compared to diabetes mellitus alone. Consistent with these findings, serum FGL2 concentrations were significantly higher in patients with T2DM (59.65 ± 9.30 ng/mL, *n* = 105) than in healthy controls (47.28 ± 6.31 ng/mL, *n* = 110; *p* < 0.001, t-test) (Figure 2B). Among the T2DM subgroups, FGL2 levels increased progressively with albuminuria severity, with patients with macroalbuminuria showing the highest levels (66.88 ± 9.05 ng/mL) compared with those with normoalbuminuria (55.37 ± 7.76 ng/mL) and microalbuminuria (60.66 ± 8.28 ng/mL; *p* < 0.001, ANOVA) (Figure 2C), indicating a strong correlation between FGL2 and renal injury. Receiver operating characteristic (ROC) curve analysis identified an optimal serum FGL2 cutoff of 54.5 ng/mL for distinguishing patients with T2DM from healthy controls (Figure 2D), supporting the potential utility of FGL2 as a noninvasive biomarker for T2DM and for the progression of diabetic nephropathy.

### Correlation between serum FGL2 and glucose and lipid metabolism

Pearson’s correlation analysis was performed to evaluate the relationship between serum FGL2 levels and metabolic parameters in all patients with T2DM (**Fig. S1**). Serum FGL2 levels were positively correlated with FBG (**Fig. S1A**), HbA1c (**Fig. S1B**), fasting insulin (**Fig. S1C**), and HOMA-IR (**Fig. S1D**), indicating a strong association with hyperglycemia and insulin resistance. Additionally, FGL2 levels were significantly positively correlated with lipid parameters, including TG (**Fig. S1E**) and TC (**Fig. S1F**), suggesting that elevated FGL2 levels are linked not only to impaired glucose metabolism but also to dyslipidemia in patients with T2DM. These findings imply that serum FGL2 reflects overall metabolic disturbance and may serve as a marker of metabolic risk in individuals with diabetes.

### Correlation between serum FGL2 and renal function and injury markers

Pearson’s correlation analysis revealed significant positive associations between serum FGL2 levels and indicators of renal function and tubular injury in patients with T2DM (Fig. S2). Specifically, FGL2 was positively correlated with BUN (Fig. S2A) and Scr (Fig. S2B) levels, reflecting a link with declining renal function. Moreover, FGL2 levels showed strong positive correlations with renal tubular injury biomarkers, including NGAL (Fig. S2C) and KIM-1 (Fig. S2D), suggesting that elevated circulating FGL2 closely parallels the severity of renal tubular damage in diabetic nephropathy. These results support the potential role of FGL2 as a circulating biomarker of renal injury in patients with T2DM.

### Correlation between serum FGL2 and inflammatory and fibrotic markers

Pearson’s correlation analysis demonstrated significant positive correlations between serum FGL2 levels and markers of inflammation and fibrosis in patients with T2DM (Fig. S3). Serum FGL2 levels were positively associated with pro-inflammatory cytokines, including TNF-α (Fig. S3A) and IL-6 (Fig. S3B), indicating a link with systemic inflammation. Additionally, FGL2 levels were strongly positively correlated with profibrotic factors, including TGF-β1 (Fig. S3C) and CTGF (Fig. S3D), suggesting that elevated FGL2 levels reflect ongoing renal fibrotic processes. These findings suggest that FGL2 may serve as a biomarker that integrates inflammatory and fibrotic responses in diabetic nephropathy.

### High glucose induces FGL2 expression in HK-2 cells

**Figure 3.**
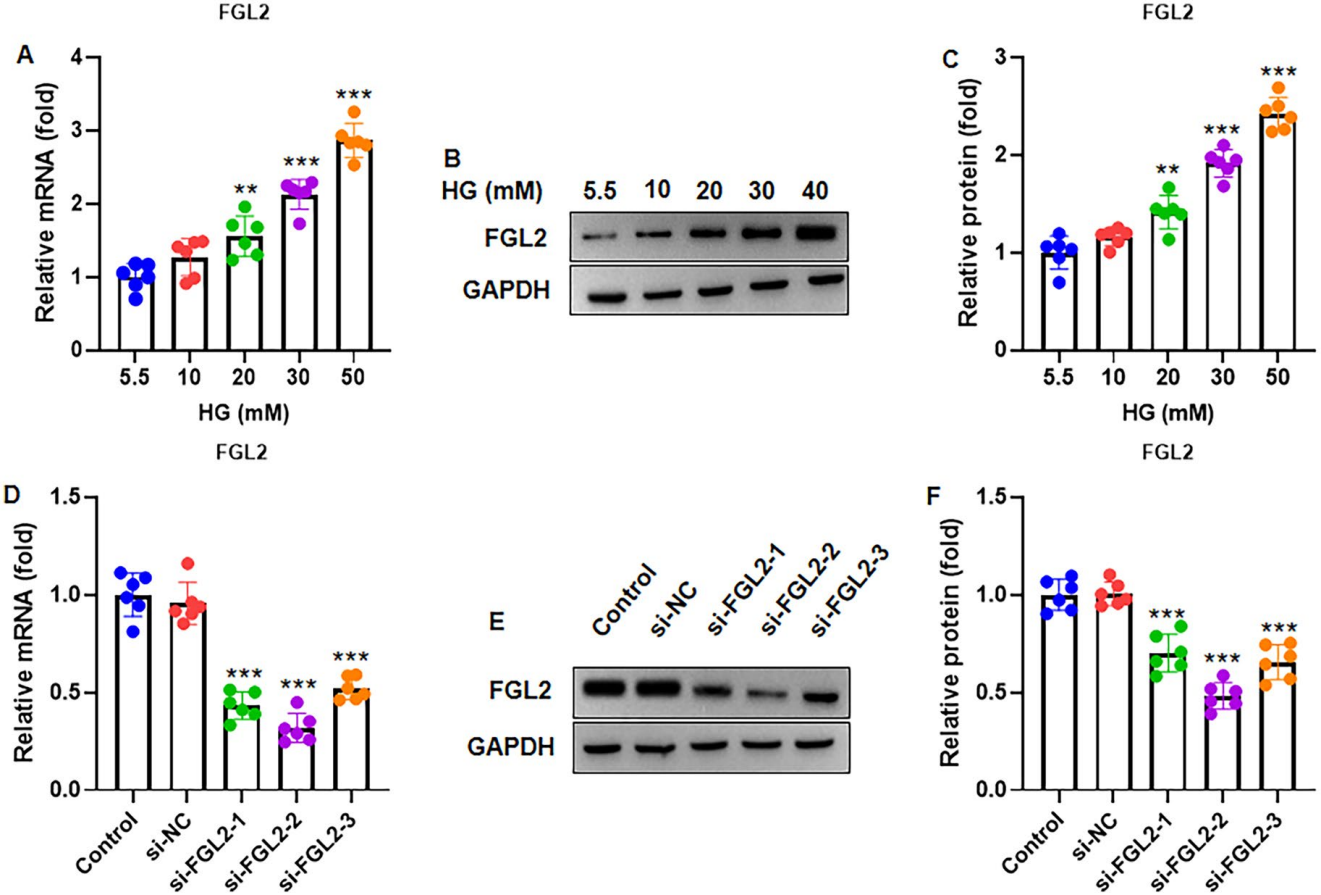
FGL2 was up-regulated in renal tubular cells treated with high glucose (HG). HK-2 cells were treated with 5.5, 10, 20, 30 and 50 mM D-glucose for 48 h. (A) The FGL2 mRNA expression was determined by RT-qPCR. (B) Representative gel blots of FGL2 by Western blot. (C) FGL2 protein blots were quantified by normalizing to GAPDH. (D) FGL2 was knocked down in HK-2 cells by transfection with three sequences of FGL2 siRNA (si-FGL2) and control siRNA (si-NC). RT-qPCR was performed to assess mRNA expression of FGL2 48 h after transfection. (E) Western blot was performed to assess protein expression of FGL2. (F) Quantification of FGL2 protein expression by normalization to GAPDH. The #2 sequence shows the best inhibitory effect, and was selected for further experiments. Data are presented as mean ± SD in triplicates. **p < 0.01, ***p < 0.001 vs control group.

Treatment of HK-2 cells with increasing concentrations of D-glucose (5.5, 10, 20, 30, and 50 mM) for 48 h resulted in dose-dependent upregulation of FGL2 at both the mRNA and protein levels. RT-qPCR analysis (Figure 3A) showed significant increases in FGL2 mRNA expression at higher glucose concentrations compared with the 5.5 mM control. Consistently, Western blot analysis (Figures 3B and **3C**) confirmed that FGL2 protein levels were elevated in a dose-dependent manner, normalized to GAPDH.

To investigate the functional role of FGL2, HK-2 cells were transfected with three different FGL2-targeting siRNA sequences (si-FGL2) or a control siRNA (si-NC). RT-qPCR (Figure 3D) and western blotting (Figure 3E and F) demonstrated that all three siRNAs reduced FGL2 expression, with sequence #2 achieving the most pronounced knockdown at both the mRNA and protein levels. Therefore, this sequence was selected for subsequent experiments. These results indicate that high glucose levels stimulate FGL2 expression in renal tubular cells and that FGL2 can be effectively silenced using siRNA for functional studies.

### FGL2 knockdown activates the PI3K/Akt pathway in high glucose-treated HK-2 cells

**Figure 4.**
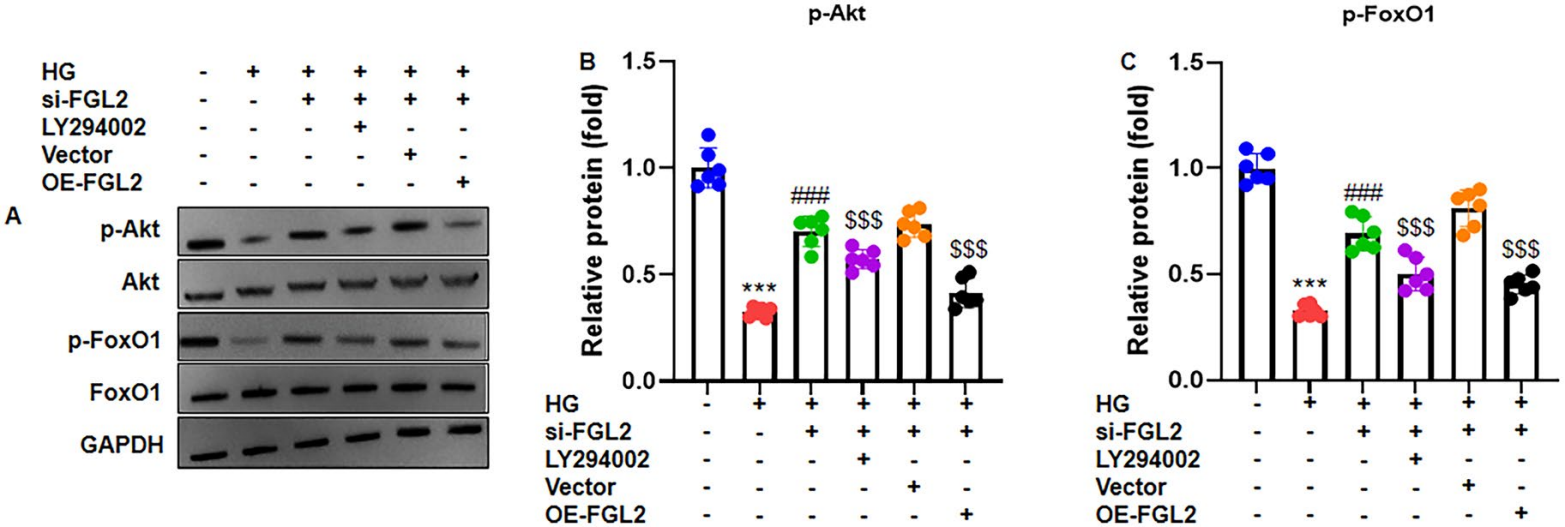
FGL2 knockdown enhances PI3K/Akt pathway in HG-treated renal tubular cells. HK-2 cells transfected with si-FGL2 or si-NC for 24 h. HK-2 cells were pretreated with LY294002 (10 μM) for 1 h, followed by exposure to high glucose (30 mM) for 24 h. (A) Representative gel blots of phosphorylated phosphorylated Akt (p-Akt, Ser473) and phosphorylated FoxO1 (p-FoxO1, Ser256). (B, C) Quantification analysis of p-Akt (normalized to total Akt) and p-FoxO1 (normalized to total FoxO1). Data are presented as mean ± SD in triplicates. ***p < 0.001 vs control group; ###*p* < 0.001 vs HG group; $$$*p* < 0.001 vs HG+si-FGL2 group.

To determine whether the effect of high glucose on the PI3K/Akt-FoxO1 pathway is mediated by FGL2, HK-2 cells were transfected with si-FGL2 or si-NC for 24 h and then exposed to high glucose, with or without pretreatment with the PI3K/Akt inhibitor LY294002 (10 μM) for 1 h. Western blot analysis showed that high-glucose stimulation activated PI3K/Akt signaling, as evidenced by increased Akt phosphorylation and altered FoxO1 phosphorylation at Ser256 (Figure 4A). Importantly, FGL2 silencing significantly modulated this response, further increasing Akt (Ser473) and FoxO1 (Ser256) phosphorylation compared with high-glucose controls. Quantitative analyses confirmed significant elevations in the p-Akt/total Akt and p-FoxO1/total FoxO1 ratios following FGL2 knockdown (Figure 4B, C). These effects were completely abolished by LY294002, confirming that the observed signaling changes are dependent on PI3K/Akt activity. Functionally, FGL2 silencing ameliorated high-glucose–induced tubular injury, as reflected by improved cell viability, reduced apoptosis, decreased oxidative stress, and attenuated extracellular matrix accumulation. Collectively, these findings demonstrate that high glucose regulates the PI3K/Akt–FoxO1 pathway in renal tubular cells in an FGL2-dependent manner, and that the protective effects observed upon FGL2 silencing are mediated through modulation of this signaling axis.

### FGL2 knockdown promotes cell viability and inhibits apoptosis in high glucose-treated HK-2 cells *via* the PI3K/Akt pathway

**Figure 5.**
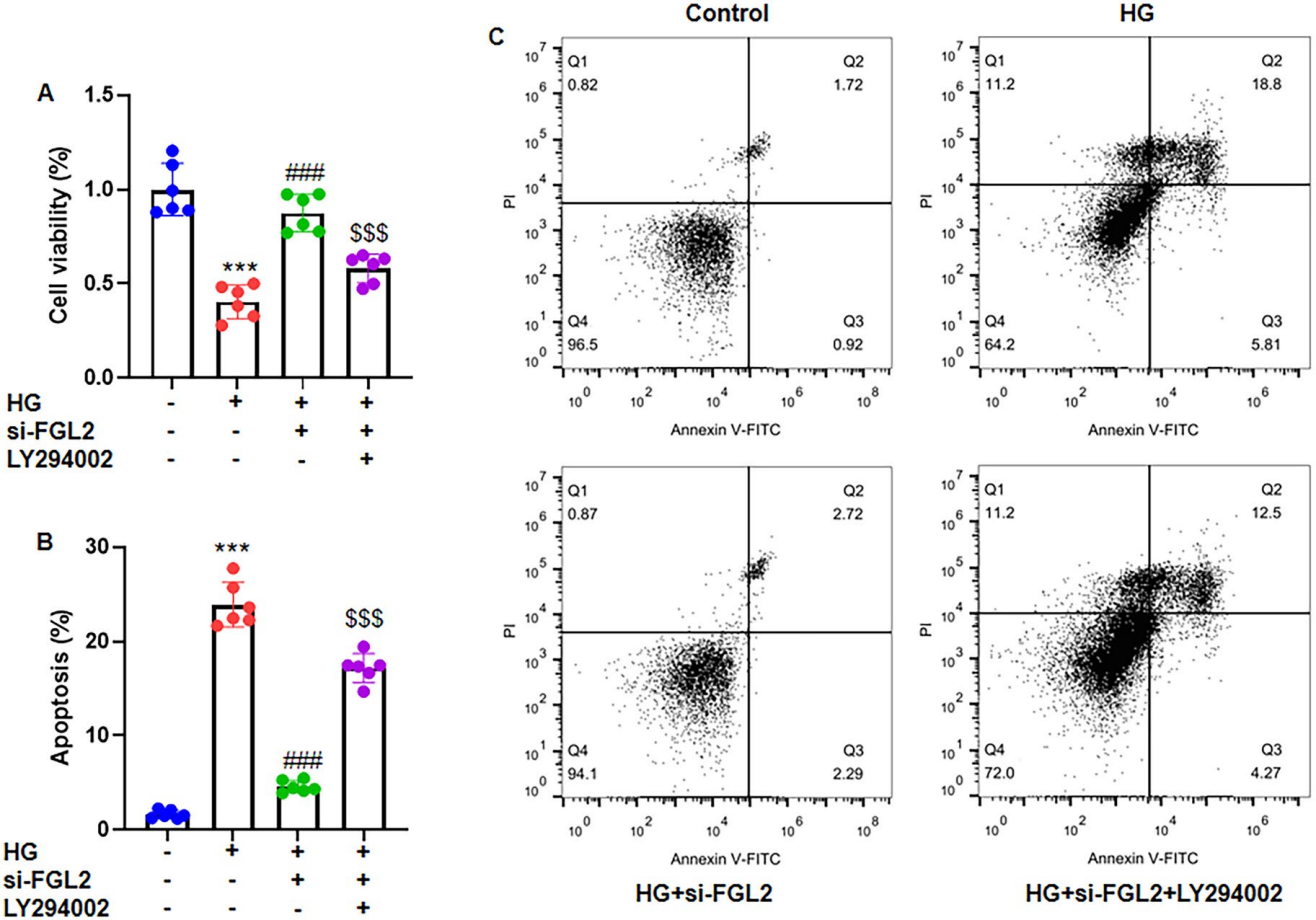
FGL2 knockdown enhances promotes cell viability and suppresses apoptosis of HG-treated renal tubular cells *via* PI3K/Akt pathway. (A) Cell viability was assessed by MTT assay. (B) Cell apoptosis was assessed by Annexin V-FITC double staining and analyzed by flow cytometry. The apoptotic rate was calculated by summation of early apoptotic cells (lower right quadrant) and late apoptotic cells (upper right quadrant). (C) Representative images of apoptosis from flow cytometry. Data are presented as mean ± SD in triplicates. ***p < 0.001 vs control group; ###*p* < 0.001 vs HG group; $$$*p* < 0.001 vs HG+si-FGL2 group.

To determine whether FGL2 mediates high glucose–induced renal tubular cell injury, HK-2 cells were exposed to 30 mM glucose following transfection with si-FGL2 or si-NC. MTT assays showed that high glucose markedly reduced cell viability, whereas silencing FGL2 significantly restored viability compared to si-NC-transfected cells, indicating that FGL2 contributes to high glucose–induced cytotoxicity (Figure 5A). Consistently, Annexin V-FITC/PI flow cytometry analysis demonstrated that high glucose–induced apoptosis was significantly prevented by FGL2 knockdown, suggesting that FGL2 mediates the pro-apoptotic effects of high glucose (Figures 5B and C). Moreover, pharmacological inhibition of PI3K/Akt signaling with LY294002 abolished the anti-apoptotic and pro-survival effects conferred by FGL2 silencing. These findings indicate that FGL2 is a key mediator of high-glucose–induced apoptosis in HK-2 cells, and that its knockdown protects against tubular cell injury by activating the PI3K/Akt pathway.

### FGL2 knockdown prevents high glucose-induced ROS accumulation by relieving inhibition of the PI3K/Akt pathway in HK-2 cells

**Figure 6.**
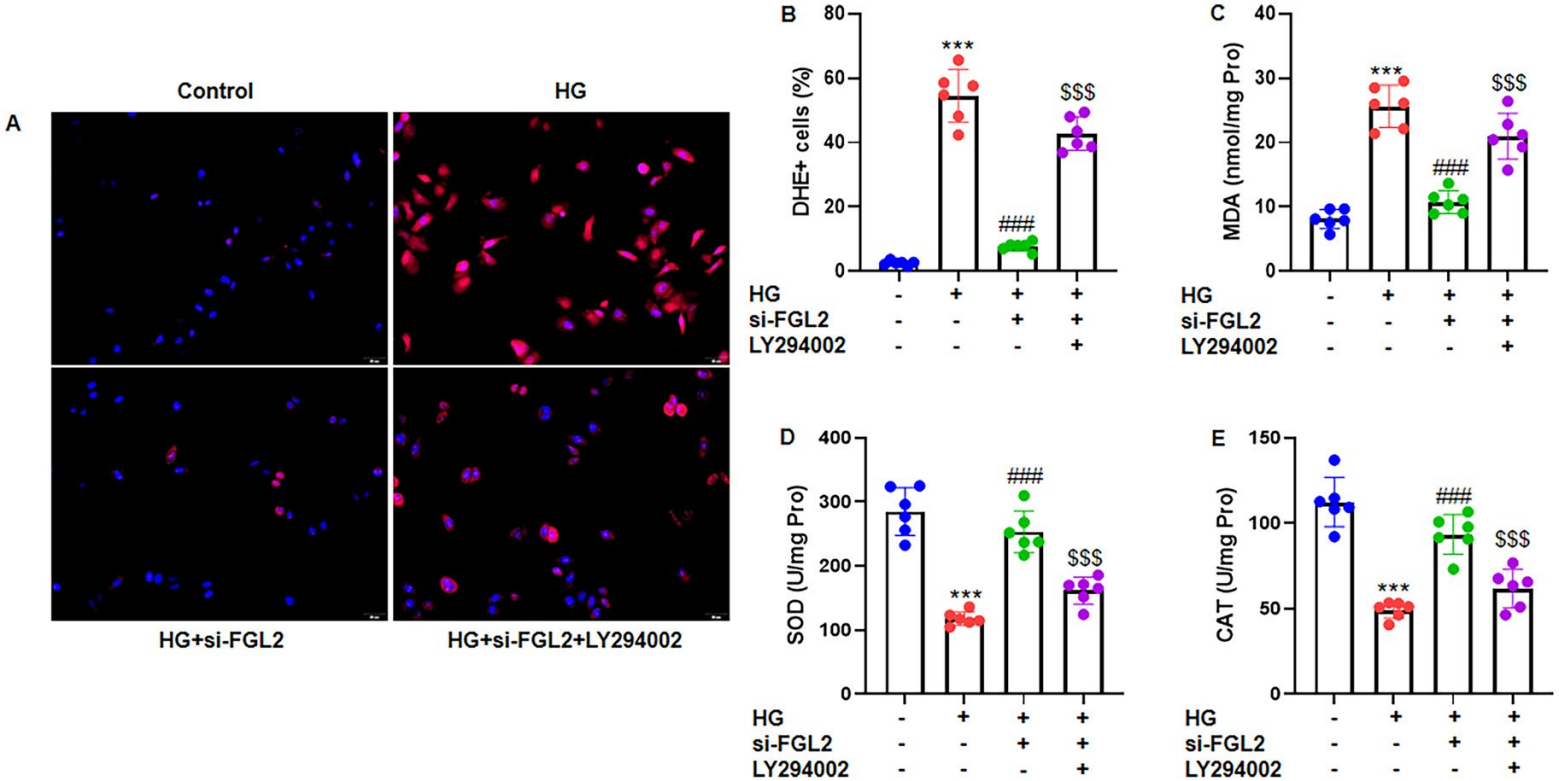
FGL2 knockdown suppresses cellular ROS production and oxidative stress in HG-treated renal tubular cells *via* PI3K/Akt pathway. (A) Representative images of DHE staining of HK-2 cells. (B) Quantification of DHE positive cells relative to DAPI positive cells. The oxidative stress indicators in lysate of HK-2 cells were measured for (C) MDA, (D) SOD and (E) CAT. Data are presented as mean ± SD in triplicates. ***p < 0.001 vs control group; ###*p* < 0.001 vs HG group; $$$*p* < 0.001 vs HG+si-FGL2 group.

The effects of FGL2 on oxidative stress in HK-2 cells under high-glucose conditions were assessed. DHE staining showed that high glucose markedly increased intracellular ROS levels, whereas silencing FGL2 prevented the high glucose-induced increase in ROS, as evidenced by a reduced proportion of DHE-positive cells relative to DAPI-positive cells (Figures 6A and B). Consistently, biochemical analyses demonstrated that FGL2 knockdown attenuated oxidative stress induced by high glucose, as indicated by decreased MDA levels (Figure 6C) and increased activities of the antioxidant enzymes SOD (Figure 6D) and CAT (Figure 6E) compared with si-NC-transfected cells under high-glucose conditions. Importantly, pharmacological inhibition of PI3K/Akt signaling with LY294002 abolished the antioxidant and cytoprotective effects conferred by FGL2 silencing, indicating that FGL2 suppresses PI3K/Akt signaling under high-glucose conditions, and that knockdown of FGL2 alleviates oxidative stress by releasing this inhibitory effect on the PI3K/Akt pathway.

### FGL2 knockdown regulates ECM-related genes and proteins *via* the PI3K/Akt pathway

**Figure 7.**
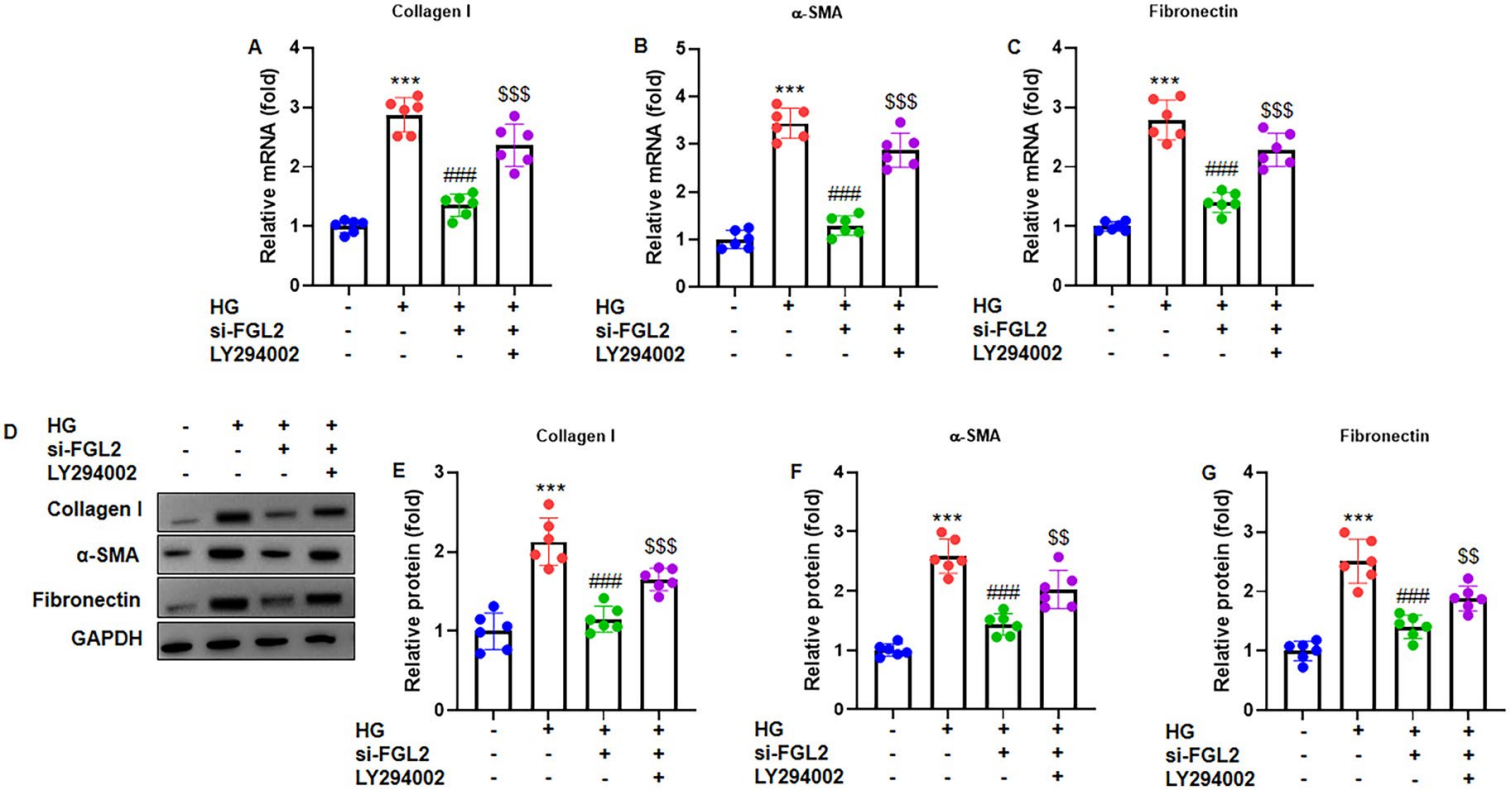
FGL2 knockdown modulates ECM-related genss *via* PI3K/Akt pathway. RT-qPCR was used to assess mRNA expression of (A) Collagen I, (B) α-SMA and (C) Fibronectin. (D) Western blot was performed to determine ECM-related proteins. Quantification of (E) Collagen I, (F) α-SMA and (G) Fibronectin protein, which were normalized to GAPDH. Data are presented as mean ± SD in triplicates. ***p < 0.001 vs control group; ###*p* < 0.001 vs HG group; $$$*p* < 0.001 vs HG+si-FGL2 group.

To investigate the role of FGL2 in extracellular matrix (ECM) remodeling under high-glucose conditions, HK-2 cells were transfected with si-FGL2 or si-NC and treated with 30 mM glucose. RT-qPCR analysis (Figure 7A–C) showed that FGL2 knockdown significantly reduced the mRNA expression of Collagen I, α-SMA, and fibronectin compared with the si-NC controls. Consistently, Western blot analysis (Figure 7D–G) demonstrated that the protein levels of Collagen I, α-SMA, and fibronectin were markedly decreased following FGL2 silencing, normalized to GAPDH. These inhibitory effects were partially reversed by the PI3K/Akt inhibitor LY294002, indicating that FGL2 regulates ECM-related gene and protein expression in high-glucose-treated renal tubular cells *via* the PI3K/Akt signaling pathway.

## Discussion

In this study, we demonstrated for the first time that serum FGL2 levels are significantly higher in patients with T2DM than in healthy controls, and that levels rise progressively with the severity of albuminuria. At the cellular level, high-glucose exposure upregulated FGL2 in HK‑2 tubular cells, whereas FGL2 knockdown enhanced PI3K/Akt signaling (increased p‑Akt and p‑FoxO1), improving cell viability, reducing apoptosis and oxidative stress, and suppressing ECM expression. Collectively, these findings suggest that FGL2 may acts as both a biomarker and a mechanistic mediator of tubular injury in diabetic nephropathy *via* the Akt/FoxO1 axis.

Although our data show that FGL2 is an adverse regulator of the Akt/FOXO1 signaling pathway, the upstream mechanisms by which FGL2 represses PI3K/Akt activities remain poorly understood. Non-renal systems are beginning to provide evidence that FGL2 might suppress Akt signaling, which is relevant to diabetic kidney disease (DKD) [7,16]. (i) Soluble FGL2 (sFGL2) has been reported to stimulate the secretion of proinflammatory cytokines (TNF-α, IL-6), which inhibits PI3K/Akt signaling through insulin resistance and stress-induced activation of the kinases [7,17,18]. Exposure to chronic cytokines represses tubular Akt signaling and is associated with DKD development [19,20], implying that FGL2 may indirectly suppress Akt/FoXO1 *via* inflammatory amplification. (ii) Membrane-bound FGL2 has prothrombinase activity and leads to endothelial dysfunction and microvascular ischemia [21]. Akt suppression in renal tubules is mediated by endothelial stress, hypoxia, and impairments in tubular-endothelial crosstalk [22–24], suggesting that FGL2 inhibits Akt *via* a vascular pathway. (iii) FGL2 can also control intracellular stress signals, such as oxidative stress, ER stress, and phosphatase activity, like PTEN, which are all detrimental to PI3K/Akt signaling [7,25]. Such stresses stimulate Akt dephosphorylation and FoxO1 activation in DKD [1,2]. Taken together with the previous results, we suggest that FGL2 inhibits the PI3K/Akt-FoxO1 pathway through the concerted action of inflammatory, vascular, and intracellular stress-induced mechanisms, rather than through a direct molecular interaction. Further research employing *in vivo* diabetic models, receptor-blocking interventions, and phosphatase-specific measurements will be necessary to establish the upstream signaling pathways through which FGL2 mediates Akt inhibition in diabetic nephropathy.

Compared with established tubular injury biomarkers, such as NGAL and KIM‑1, which are effective in detecting early tubular stress but do not fully capture systemic inflammatory or profibrotic processes that drive disease progression, FGL2 may provide complementary information by reflecting both systemic and renal microenvironmental perturbations. Current tubular markers primarily indicate structural stress responses but lack the capacity to report broader immunomodulatory or coagulation-related signaling cascades implicated in diabetic kidney disease (DKD) progression [26–28]. Similarly, albuminuria remains the clinical standard for staging DKD; however, it often becomes detectable only after substantial tubular injury has occurred and exhibits limited sensitivity for the earliest stages of renal damage [29–31].

Our clinical data demonstrate a robust elevation of serum FGL2 levels in patients with T2DM compared to healthy controls, with a stepwise increase corresponding to the severity of albuminuria. This pattern suggests a close association between circulating FGL2 and renal injury burden in diabetic nephropathy. Recent transcriptomic studies have consistently shown that renal FGL2 mRNA expression is upregulated in CKD and correlates with fibrosis and adverse renal outcomes [6]. In our cohort, serum FGL2 levels were significantly correlated with established markers of tubular injury (NGAL, KIM-1), renal dysfunction (blood urea nitrogen, serum creatinine), metabolic dysregulation (fasting blood glucose, HbA1c, HOMA-IR, triglycerides, and total cholesterol), inflammatory mediators (TNF-α and IL-6), and profibrotic factors (TGF-β1 and CTGF), supporting its role as an integrative indicator of metabolic, inflammatory, and tubular pathological processes in DKD. ROC curve analysis (cutoff value, 54.5 ng/mL) further suggested potential diagnostic utility for distinguishing diabetic from non-diabetic individuals. However, given the cross-sectional design of the clinical component, these associations do not establish a temporal sequence or causality. Therefore, serum FGL2 should currently be interpreted as a biomarker reflecting ongoing disease activity rather than a predictor of future disease onset or progression, underscoring the need for prospective validation studies.

Importantly, circulating FGL2 may not be exclusively derived from tubular epithelium. FGL2 is expressed by activated macrophages, T cells, endothelial cells, and other immune populations, where it exerts immunomodulatory and prothrombinase activities [32]. Elevated serum FGL2 has been reported in systemic inflammatory conditions, including sepsis, viral infections, and autoimmune diseases, often paralleling levels of cytokines and coagulation markers [7,33]. In CKD, higher FGL2 levels correlate with systemic inflammation and coagulation, suggesting that circulating levels reflect both renal and extra renal immune activation.

Although *in vitro* experiments provide mechanistic insights into the role of FGL2 in tubular epithelial responses to hyperglycemic stress, diabetic nephropathy is a complex and heterogeneous disease that cannot be fully modeled using a single cell line. DN progression involves coordinated interactions among glomerular cells, tubular epithelial cells, endothelial cells, immune cells, and systemic metabolic and inflammatory factors, all of which contribute to renal dysfunction and fibrosis [3,4,34]. In this context, the HK-2 cell system represents a reductionist model designed to isolate the direct effects of high glucose and FGL2 signaling on proximal tubular epithelial cells, which are now recognized as early and independent drivers of DKD progression [3,5].

From a translational perspective, serum FGL2 may represent a noninvasive marker reflecting tubular injury and profibrotic activity in patients with T2DM. Its strong associations with metabolic dysregulation, inflammatory burden, and fibrotic indices support its potential utility for disease characterization and risk stratification rather than prediction. However, longitudinal cohort studies are required to determine whether elevations in circulating FGL2 precede the onset of diabetic nephropathy or are independently associated with adverse renal outcomes. In parallel, mechanistic findings suggest that FGL2 contributes to tubular dysfunction through Akt/FoxO1 signaling, raising the possibility that modulation of this pathway could confer tubular protection in diabetes. Given the stepwise increase in FGL2 levels with worsening albuminuria, future interventional studies targeting FGL2 may be particularly relevant in the early or progressive stages of diabetic nephropathy.

### Limitations

This study has several limitations that should be acknowledged. First, the clinical component was cross-sectional in design, which precludes causal inference and does not allow the assessment of prognostic or predictive value. Although serum FGL2 levels were strongly associated with the presence and severity of diabetic nephropathy, prospective longitudinal studies are required to determine whether FGL2 can predict incident disease, renal function decline, or progression to end-stage kidney disease. Second, the mechanistic investigations were confined to HK-2 proximal tubular epithelial cells exposed to high-glucose conditions. While this *in vitro* model is well established for examining tubular responses relevant to diabetic kidney disease, it does not recapitulate the full multicellular and hemodynamic complexity of diabetic nephropathy, including glomerular–tubular crosstalk, immune–vascular interactions, and systemic influences. Therefore, the mechanistic conclusions derived from these experiments should be interpreted as supportive rather than definitive, and *in vivo* validation in diabetic animal models and studies using primary human tubular cells or multicellular/organoid systems are warranted. Third, although the Akt/FoxO1 axis was implicated as a downstream pathway, the precise upstream mechanisms by which FGL2 modulates Akt signaling remain unclear. It is unknown whether these effects are mediated through immunomodulatory functions, procoagulant activity, or receptor-dependent signaling in tubular epithelial cells. Fourth, a parallel osmotic control group (e.g., mannitol-treated cells) was not included in the experimental design. Future studies should incorporate such controls to further strengthen the specificity of glucose-mediated effects. Finally, prior reports of aggravated fibrosis in FGL2-deficient models of obstructive injury suggest that the role of FGL2 may be context dependent, underscoring the need for careful delineation of its function across different forms of renal pathology, including metabolic versus non-metabolic injury.

## Conclusion

In conclusion, this study identified serum FGL2 as a circulating marker associated with the severity of renal tubular injury in patients with T2DM and provided mechanistic evidence that FGL2 can directly modulate high-glucose-induced tubular epithelial dysfunction *via* the PI3K/Akt–FoxO1 signaling pathway. Although the *in vitro* findings were derived from a single tubular cell line and should be interpreted with this limitation in mind, they offered biologically plausible mechanistic support for the clinical associations observed. Further *in vivo* and multicellular studies are warranted to fully define the role of FGL2 in the pathogenesis and progression of diabetic nephropathy.

## Supplementary Material

Figure_S1 R2.TIF

Figure_S2 R2.TIF

Figure_S3 R2.TIF

Supplementary Table 1_revised R2.doc

## Data Availability

The datasets used/analyzed during the present study are available from the corresponding author upon reasonable request.
