# Supplementary figures and images for: Serum fibrinogen-like protein 2 is associated with diabetic nephropathy severity and modulates high glucose-induced tubular dysfunction via Akt-FoxO1 signaling
Source: Ren Fail. 2026 May 20;48(1):2672189. doi: 10.1080/0886022X.2026.2672189 (PMC13195712; doi:10.1080/0886022X.2026.2672189)

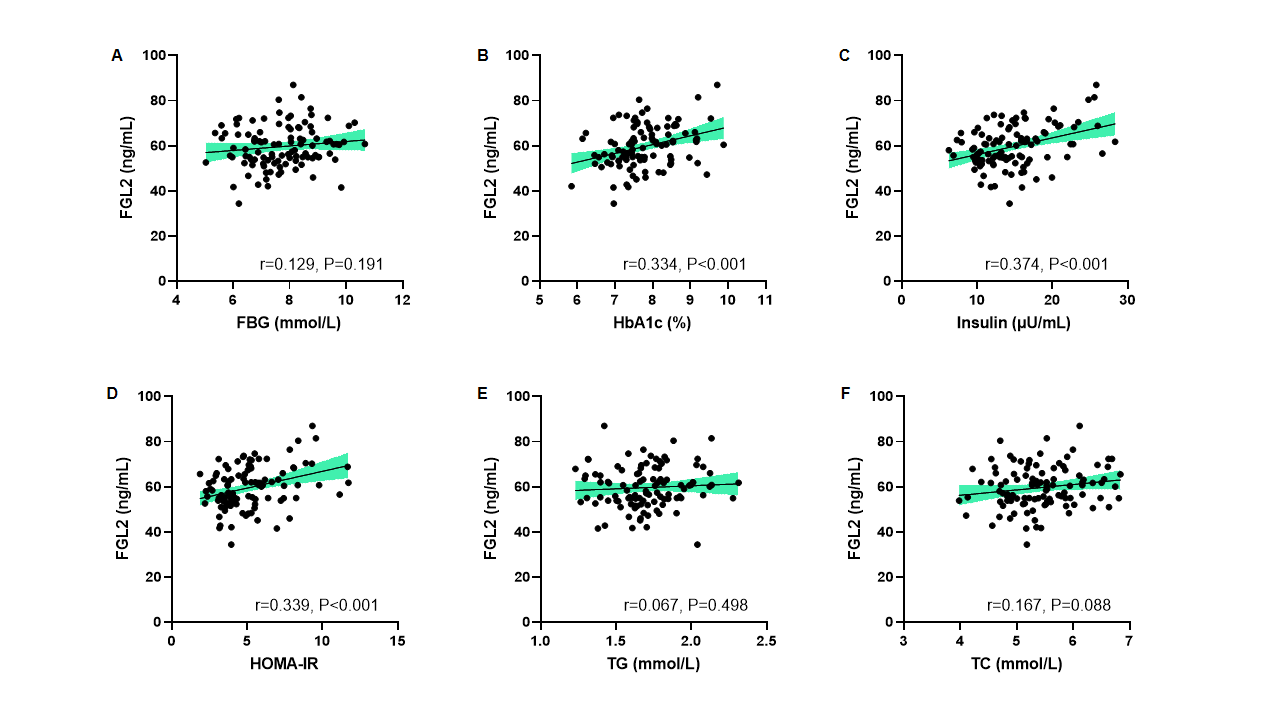

Supplement: Figure_S1 R2.TIF [file IRNF_A_2672189_SM8966.tif]

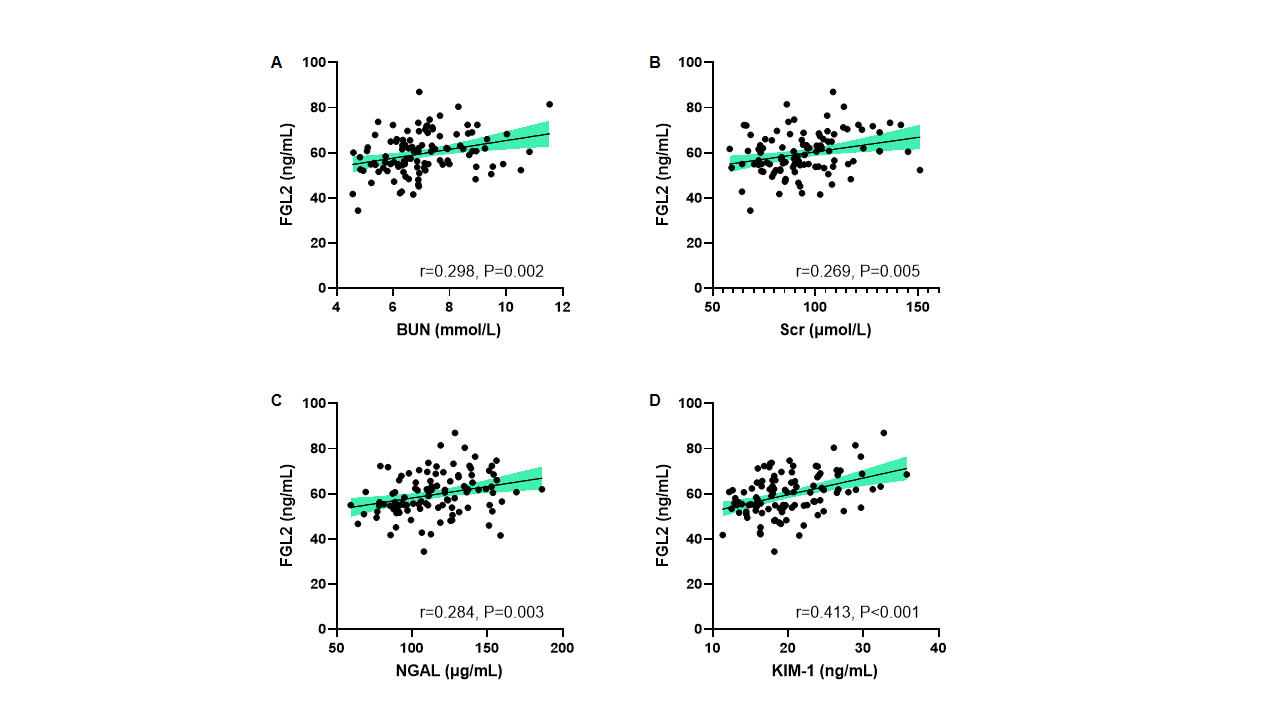

Supplement: Figure_S2 R2.TIF [file IRNF_A_2672189_SM8965.tif]

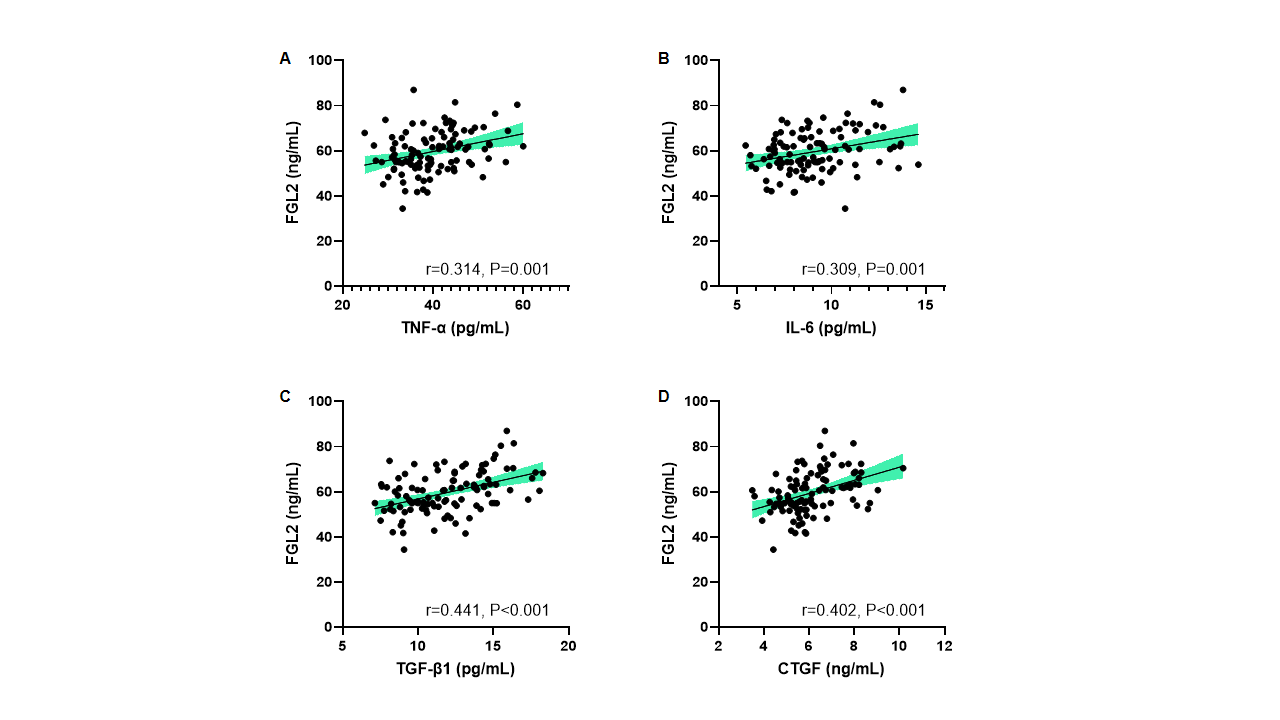

Supplement: Figure_S3 R2.TIF [file IRNF_A_2672189_SM8964.tif]
