# Supplementary material for: Serum fibrinogen-like protein 2 is associated with diabetic nephropathy severity and modulates high glucose-induced tubular dysfunction via Akt-FoxO1 signaling
Source: Ren Fail. 2026 May 20;48(1):2672189. doi: 10.1080/0886022X.2026.2672189 (PMC13195712; doi:10.1080/0886022X.2026.2672189)
Supplement: Supplementary Table 1_revised R2.doc [file IRNF_A_2672189_SM8963.doc]

**Supplementary Table 1.** List of primer sequences used in this investigation.

| **Genes** | **Forward primer (5′-3′)** | **Reverse primer (5′-3′)** |
| --- | --- | --- |
| Human FGL2 | CCAAGCACTTTAAGCCATAAATC | GGAATTAATTGCCCTATTAGATAACG |
| Human Collagen I | GAGGGCCAAGACGAAGACATC | CAGATCACGTCATCGCACAAC |
| Human α-SMA | AAAGCAAGTCCTCCAGCGTT | TTAGTCCCGGGGATAGGCAA |
| Human Fibronectin | GATACCATCATCCCAGCTGTTC | CAGGAAGTTGGTTAAATCAATGGA |
| Human GAPDH | CATGTTGCAACCGGGAAGGA | CGCCCAATACGACCAAATCAG |
